# Ultrasonic Sensor Fusion Inverse Algorithm for Visually Impaired Aiding Applications

**DOI:** 10.3390/s20133682

**Published:** 2020-06-30

**Authors:** György Kovács, Szilvia Nagy

**Affiliations:** Széchenyi István University, Department of Telecommunications, Egyetem tér 1 Hungary, H9026 Győr, Hungary; nagysz@sze.hu

**Keywords:** ultrasound echolocation, depth mapping, sensor fusion, time of flight, visually impaired

## Abstract

Depth mapping can be carried out by ultrasound measuring devices using the time of flight method. Ultrasound measurements are favorable in such environments, where the light or radio frequency measurements can not be applied due to the noise level, calculation complexity, reaction time, size and price of the device, accuracy or electromagnetic compatibility. It is also usual to apply and fusion ultrasound sensors with other types of sensors to increase the precision and reliability. In the case of visually impaired people, an echolocation based aid for determining the distance and the direction of obstacles in the surroundings can improve the life quality by giving the possibility to move alone and individually in unlearnt or rapidly changing environments. In the following considerations, a model system is presented which can provide rather reliable position and distance to multiple objects. The mathematical model based on the time of flight method with a correction: it uses the measured analog sensor signals, which represent the probability of the presence of an obstacle. The device consists of multiple receivers, but only one source. The sensor fusion algorithm for this setup and the results of indoor experiments are presented. The mathematical model allows the usage, processing, and fusion of the signals of up to an infinite number of sensors. In addition, the positions of the sensors can be arbitrary, and the mathematical model does not restrict them to be placed in regular formations.

## 1. Introduction

The largest part of the information from our environment is collected usually by our sights. However, in the case of lack or damaged sight, the other senses become emphasized. A part of the visually obtainable information thus can be gained by other means, but the visually handicapped people are still restricted in their movements in such places, which are not known to them.

In the past, several ideas have already been realized to help the navigation of the visually impaired people. The well known and commonly used methods are the white cane and/or the service dog. Thanks to the level of the technology, new methods were developed to give feedback to the user about the environment. Most of the solutions use cameras, to recognize defined objects or read text or to make a depth map [1,2,3,4,5]. The feedback is in most cases an audio feedback, like a warning sound or the system loudly reading the recognized text. The sound is one of the most important input channels if the sight is not functioning fully.

The camera solutions are mostly used in indoor environments because the changing of the light can cause disorder in the image processing. This disorder can lead to measuring ghost objects or not detecting existing objects. In an outdoor environment, the cameras are rarely usable or usable only with special additional systems—IR lighting and IR camera. The camera functions as the human eye, and it is able to replace the sight. However, computational image processing is still far away from the possibilities from a human brain. Ultrasound distance measurement has the advantage that it is not that much affected by environmental changes—by the change of the air density, humidity, etc., the accuracy will not change so dramatically that the measurement would not be acceptable, and the processing of the signals does not need that high a level of calculation resources. The shortcomings of gaining the information from ultrasound measurements is not giving color feedback, and the depth map not being that detailed in every case as the visual solutions. As the resolution of the ultrasound measurements is mostly lower than the optical solutions, they can be used for bigger distances.

The aim of the present research is to develop equipment to visually handicapped people that partially substitutes the sight by providing information about the obstacles in the environment. The device is a goggles or headband-like object with multiple sensors and one source. Modified time-of flight method was applied with a sensor fusion algorithm for determining the position of the obstacles in the environment. Later, this instrument can be attached to an acoustic signal generator and a headset that can give warnings, or continuous information about objects in the direction the source and sensors are facing at a sensing angle of about 40 degrees.

In the following article, the possibilities of the 3D environment mapping will be summarized in Section 2. Then, in Section 3, the theoretical ideas of the sensor fusion algorithms are summarized. Next, in Section 4, the mathematical model is presented, and, as a last step, the implemented system is tested and the results are given in Section 5.

## 2. Measurement Methods for Object Localization

3D object localization is a very important task in many fields of everyday life as well as research and development. Ultrasonic distance sensing is one of the key possibilities in many localization solutions.

For industrial, automotive as well as for home applications up to date solutions exist for scanning, mapping the real environment to a virtual one. In an industrial environment, a very important application, for example, is the safety programming of robots, where the operator works together with a robot, or there is a chance that a human enters the working space of the robot. There are some robots that need to recognize the solid objects in the nearby area, in order to determine whether they can move without having a collision. Nowdays, the cars have different depth scanning sensors, for example, for driving or parking assistance purposes [6].

Nowadays, in the time of virtual reality—VR— learning and working, gaming, various companies have solutions for placing the real coordinates of the users into a VR coordinate system. In the case of the VR, working or gaming a solution to measure and map the environment is also needed. Besides robotics, automotive, and gaming, there are industrial and safety systems that are also using ultrasonic tracking in smaller—5 m—distance [7,8,9,10,11].

Environment mapping has more possibilities. Usually, depth mapping solutions use more types of methods; then, with a fusion calculation, the result exploits the strengths of each method. The trend nowadays is to measure the position of solid objects with optical methods (such as stereo image processing, lidar) or radio frequency (radars or triangulation) or ultrasonic echolocation.

There are two main models for 3D localization measurement, the first is to measure the phase offset of the reflected signal, which is mainly used in the case of optical measurements [12], while the second is to base the measurement on the time-of-flight principle (TOF), which is preferred in ultrasonic measurements. In the following sections, these TOF ultrasonic measurements will be discussed.

### 2.1. 1D Measurement

One of the oldest and simplest possible methods for distance measurement is TOF. If a transmitter sends out a ping—a short ultrasonic impulse signal—it will be reflected from the solid objects, thus the distance is calculated from the traveling time of the sound and its echo. The time between the ping and the signal detection gives the traveling time. A simple multiplication with the speed of the sound can give the Distance Of Flight (DOF) [13], the resulting formula for the distance *s*, using the velocity of the sound c≈340 m/s, and the travel time *t* is

(1) s=c·t.

**Figure 1:**
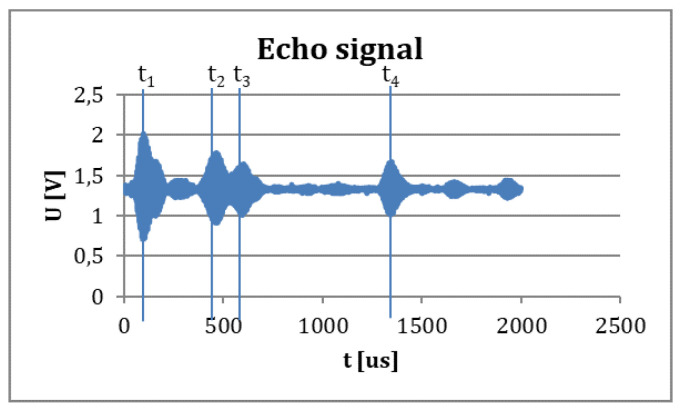
A measured echo signal in a one-sensor ultrasound measurement. The detected four objects’ four traveling times are marked (t1, t2, t3, t4) on the measured signal.

A typical measured signal with four obstacles can be seen in Figure 1.

### 2.2. 2D Measurement

For measuring in more dimensional spaces, a sensor array (vector receiver, consisting multiple sources and receivers) is needed. For the determination of the distances, there are several combinations and calculation possibilities; the most common one is to calculate with angles.

**Figure 2:**
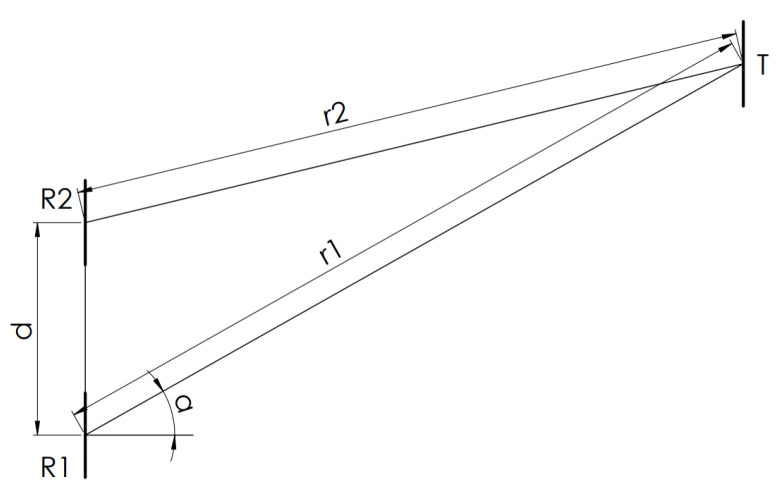
A direct measurement with one transmitter and a two-element vector receiver [13].

In order to demonstrate the calculation of the angles, based on [13], as a first step, the method is given for determining the angles in the case of a direct measurement with one transmitter and two receivers. From Figure 2, it is easy to give the trigonometrical formulae for the angles in the measurement.

To calculate the angle between the directly transmitted signals and the receivers, Formula (2) gives us the result,

(2) $$\alpha =\frac{\pi }{2}-\arccos\left(\frac{r_{1}^{2}-r_{2}^{2}+d^{2}}{2r_{1}d}\right).$$

**Figure 3:**
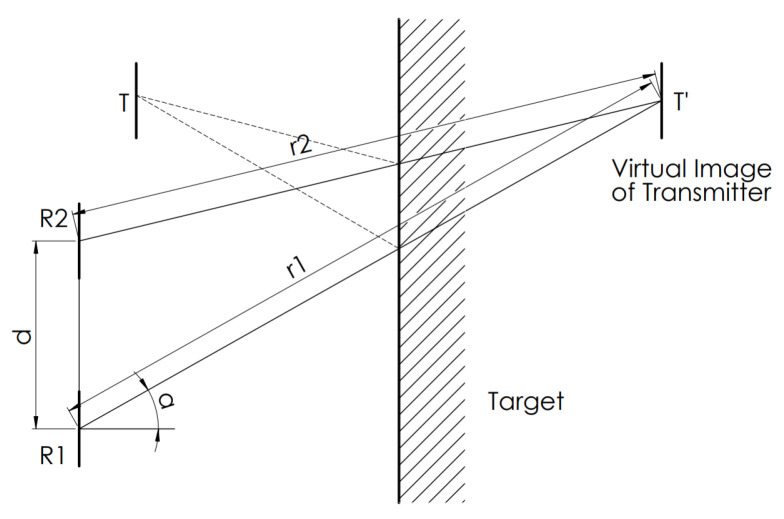
Indirect measurement setup according to [13].

To use these considerations for an indirect measurement—the reverberation of transmitted signal is sensed by the receivers, where the echo was reflected from the measured object—the transmitter has to be located according to Figure 3. The calculation is similar to the direct measurement, only the transmitter position is changed, resulting in Formula (2).

**Figure 4:**
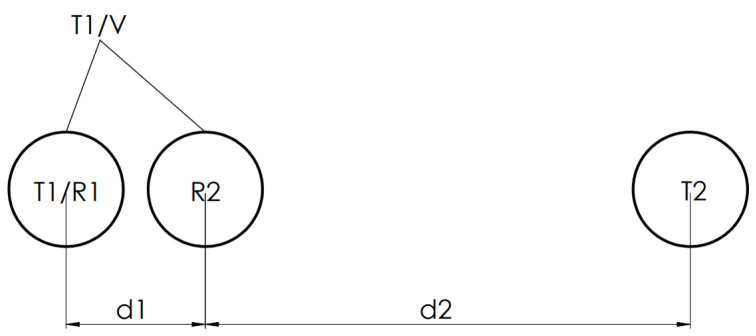
Sensor configuration [14].

To measure more complex objects in 2D, the sensor array has to be changed (like e.g., in Figure 4) because the information from the previous 2-element array is not sufficient to differentiate between the planes, edges, or corners [14].

### 2.3. 3D Measurement

**Figure 5:**
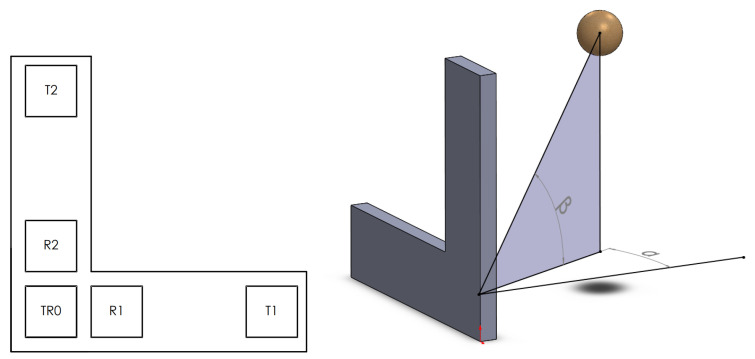
Sensor array example for 3D measurement [13].

For 3D detection, the already used vector receiver should be developed further to get a measurable third dimension, visible to the one given in the first part of Figure 5. The calculation is the same as in the 2D case; the vector receiver—TR0, R1, T1—detects the localization angle in the horizontal view and the vector receiver—TR0, R2, T2—is responsible for the vertical angle, as shown in the second subplot of Figure 5.

## 3. Fusion

As the signals of the multiple sensors have to be processed in the manner that they would give plausible results, a sensor fusion algorithm has to be applied.

Positioning and mapping systems use various methods to perform a fusion in an over-defined system to have the most accurate measurement result [15,16,17,18], but a fusion has the disadvantage that it needs to be calculated mostly by complex and computationally demanding methods, like Kalman filter or sensor covariance calculations [6,19,20]. For a *d* dimensional case, the number of necessary receivers is *d* and, if the number is less, the system becomes underdefined, while, if the receivers’ number is larger than *d*, the system is overdefined.

If there are more transmitters in the system, the signals cannot be easily separated by signal processing. A commonly used solution for avoiding the processing of combination of multiple signals is to ping from the transmitters after each other, or to use signals with different modulation simultaneously. Kasami codes are used for modulating or coding the signals, to be able to mark and identify the different sources, so that the transmission would be possible at the same time [15,16].

Typically, ultrasound measurement alone is not used as a solution for mapping or tracking because it works in a limited environment [21]. The reason for this fact is that in echolocalization the signal might have some interference with itself if the environment or the system changes in time. Similar problems exist if the distances are too small or too big compared to the size objects [22].

Often, the sensor arrays are placed symmetrically, as the resulting mathematic formulae are that way simpler and, with such distribution of the sensors, the calculation requirement is also reducible [23,24,25,26]. However, based on the system geometry, it is not always possible to have a symmetrical array of sensors. In these cases, the mathematic fusion algorithm has to handle both the different types of calculation methods and the different types of sensors.

## 4. Fusion of Ultrasonic Sensors

### 4.1. Creating the Matrix System for the 3D Mapping, and Calculating the Theoretical Distances for the Sensor Group

To make the fusion between the sensors easier, it is more favorable to use distances instead of angles [13,14,27,28,29]. Using distance coordinates makes it possible to add sensors to the system without changing the whole algorithm, i.e., the algorithm can be adapted and extended to the demand.

To create a 3D map from the signals of a sensor group of *I* elements, the algorithm calculates a reversed way compared to the TOF method.

From the 3D position given by the coordinates *x*, *y*, and *z*, the algorithm assigns one distance $s_{i}$ to every sensor. Because of the programming of the algorithm, the sensors need to be marked, i.e., every sensor has an index *i* with i=1…I.

**Figure 6:**
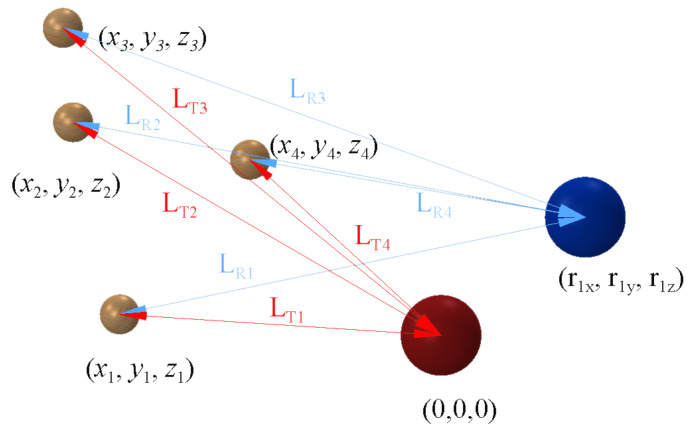
The measurement setup with one transmitter (in the origin), and one receiver plotted in blue (at position ($r_{1x},r_{1y},r_{1z}$).

The position of the transmitter can be selected as the origin (0, 0, 0), measured from here, the position $r_{ix}$, $r_{iy}$, $r_{iz}$ of every sensor is given as it can be seen in Figure 6.

From these considerations, to calculate the sensor distances for one point at x,y,z, the following result is given based on pure geometric rules:

(3) $$\begin{array}{ccc} s_{i}\;\; & = & \;\;L_{T}+L_{R}=\\ \;\; & = & \;\;\sqrt{{\left(r_{ix}-x\right)}^{2}+{\left(r_{iy}-y\right)}^{2}+{\left(r_{iz}-z\right)}^{2}}+\\ \;\; & + & \;\;\sqrt{x^{2}+y^{2}+z^{2}} \end{array}$$

To calculate the distances not only for just one point, but, for every point of a defined space, a matrix is needed S of size A×B×C, which describes the sensor distances $r_{ix}$, $r_{iy}$, $r_{iz}$ for every evaluated point $x_{a}$, $y_{b}$, $z_{c}$. For helping the algorithm to be able to move within the matrix S, the three coordinates $x_{a}$, $y_{b}$, $z_{c}$ have indices a,b,c with the ranges of a=1…A, b=1…B, c=1…C. Thus, including the sensor index *i* results in a 4D matrix,

(4) $$\begin{array}{ccc} S_{iabc}\;\; & = & \;\;\sqrt{{\left(r_{ix}-x_{a}\right)}^{2}+{\left(r_{iy}-y_{b}\right)}^{2}+{\left(r_{iz}-z_{c}\right)}^{2}}+\\ \;\; & + & \;\;\sqrt{x_{a}^{2}+y_{b}^{2}+z_{c}^{2}}. \end{array}$$

In this matrix, the indices indicate the coordinates and for these coordinates the sensor distances create the following matrix:

(5) $$S=\left[\begin{array}{cccc} S_{p}{}_{11} & S_{p}{}_{21} & \ldots & S_{p}{}_{A1}\\ S_{p}{}_{12} & S_{p}{}_{22} & \ldots & S_{p}{}_{A2}\\ \vdots & \vdots & \; & \vdots \\ S_{p}{}_{1B} & S_{p}{}_{2B} & \ldots & S_{p}{}_{AB} \end{array}\right]$$

with

(6) $$S_{p}ab=\left[\begin{array}{cccc} S_{1ab1} & S_{1ab2} & \ldots & S_{1abC}\\ S_{2ab1} & S_{2ab2} & \ldots & S_{2abC}\\ \vdots & \vdots & \; & \vdots \\ S_{Iab1} & S_{Iab2} & \ldots & S_{IabC} \end{array}\right]$$

By adjusting the ratio between the indices *a*, *b*, and *c* and the coordinates of the physical location $x_{a}$, $y_{b}$, and $z_{c}$, the raster of the mapping can be set. If the chosen ratio is 1 to 1, then the matrix can already be used as a meter—or mm—depending on the signal measurement resolution map. With the change in this ratio, the resolution can be defined higher or lower, thus the precision and the computation demand can be tuned according to the resources and the needs. In the following calculations, it will be 1 to 1.

### 4.2. Finding Amplitude with Approximation

Because of the signal measurement can be performed only with a discrete measuring equipment, the resulting amplitudes will be given only at certain time points—measuring frequency—and not continuously. If an amplitude is needed between two discrete measured points, an approximation is necessary.

For every distance, there is an amplitude from each sensor; therefore, the algorithm assigns an amplitude matrix A to distance matrix S

(7) $$\begin{array}{ccc} A\left(S_{iabc}\right)\;\; & = & \;\;A\left(\left\lfloor S_{iabc}\right\rfloor \right)+\frac{S_{iabc}-\left\lfloor S_{iabc}\right\rfloor }{\left\lceil S_{iabc}\right\rceil -\left\lfloor S_{iabc}\right\rfloor }\times \\ \;\; & \times & \;\;\left(A\left(\left\lceil S_{iabc}\right\rceil \right)-A\left(\left\lfloor S_{iabc}\right\rfloor \right)\right). \end{array}$$

Here, ⌊•⌋ and ⌈•⌉ denote the floor and ceil operations that are necessary because of the discrete measurement.

### 4.3. Reduction to Probability Variable

With the previous calculation, after the mapping system is created, the amplitudes belonging to all the sensors in every observed point are exactly known. For defining how big the probability is in an observed location of whether there is a reflective object or not, the probability matrix $A^{*}$ was introduced. To switch over from amplitude matrix A to probability matrix $A^{*}$, the probability for each sensor should be calculated using normalization with the $A_{max}$, i.e., maximal possible amplitude of the sensor according to the following formula:

(8) $$A^{*}\left(S_{iabc}\right)=\frac{A\left(S_{iabc}\right)}{A_{max}}.$$

### 4.4. Sensor Fusion

The probability can be multiplied, from all of the sensors, to have just one probability for every evaluated location of the observed area. With this reduction, the 4D matrix will decrease to a 3D matrix P

(9) $$P\left(a,b,c\right)=\frac{\prod_{i=0}^{I}A^{*}\left(S_{iabc}\right)}{I},$$

where I is the number of the used receivers.

**Figure 7:**
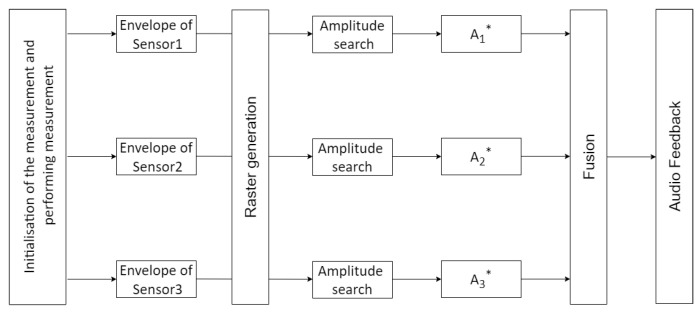
The flow chart of the algorithm.

The flowchart in Figure 7 summarizes the operation of the algorithm.

The result of the algorithm has a basic difference compared to other research [21] in which the presented calculation gives back a non-binary result of the positions, the probability of each detected obstacle.

## 5. Implementation of the Theory

The fusion of two sensors already gives a good approximation about the positions of the obstacles in the observed area; moreover, after the testing with three sensors’ fusion, the results were more detailed. While adapting the theoretical measurement to the real-life application, the sufficient number of the sensors had to be decided. As the device is planned to be wearable and affordable, this number should not be too high, maximum 3. In addition, increasing the number of sensors significantly enlarges the numerical complexity. As blind people are able to localize virtual sound sources in about 15–20${}^{{}^{\circ}}$ precision [1], the localization does not have to be more accurate than this.

**Figure 8:**
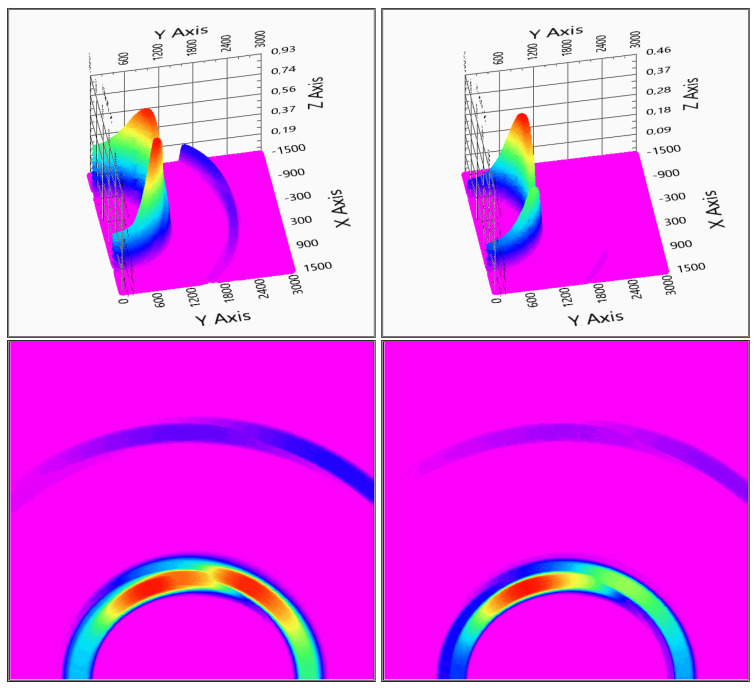
Test measurement with two and three sensors. On the left-hand side, the two sensors measurement on the right-hand side the three sensors measurement is visible. For better visuality in the bottom row, top views are also shown. (x,y: x,y space axis, *z*-axis: probability of solid object in an x,y defined position).

In the next section, an implemented system is tested with two and three sensors in order to determine whether this precision can be achieved with two sensors or the three sensors’ setup is necessary. See Figure 8. An indoor environment was used because it made the setup easier, and it gave us the conclusion that the system can work stably, thus it is deemed that the outdoor measurements not necessary.

### 5.1. Implementation into Smart-Glasses

Our measurement method can measure and recognize the field in front of the sensors, and can make a simple depth map. This depth map is a visualization of the solid object. This is the reason why it can be implemented into a system which can help blind people to get feedback from the environment from a few meters distance.

#### System Setup

Using the echolocation, the system can recognize solid objects in 3D and can give information about their ranges and positions.

**Figure 9:**
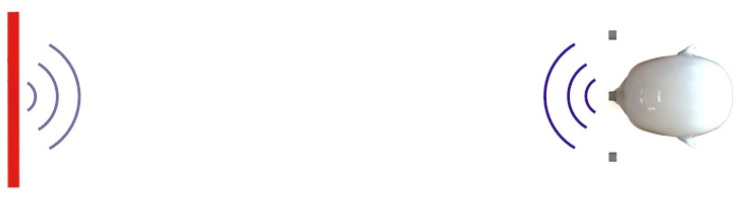
Concept of the method.

The main concept is that in the glasses there are two receivers—or three—and one transmitter, and the 125 μs long pulses at 40 kHz frequency with 50% duty cycle signals from the transmitter reflects from the solid objects as shown in Figure 9 [30].

**Figure 10:**
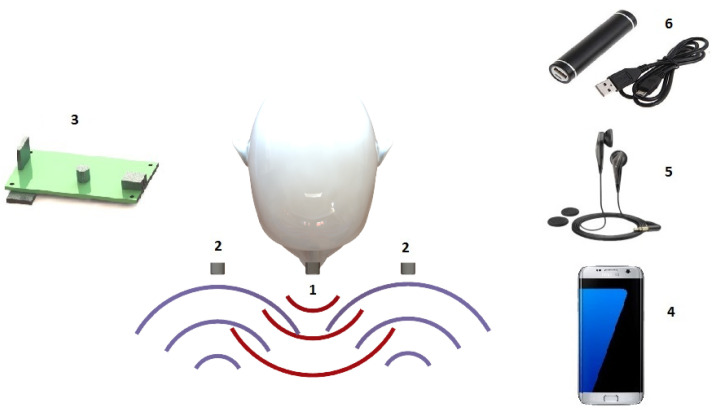
Main parts of the system: 1–transmitter, 2–receivers, 3–logic unit, 4–smart phone, 5–headphone, 6–power source.

Figure 10 gives the main parts of the system. The signals from the transmitter (1) are measured with the two receivers (2). Getting the whole system to work needs a logic unit (3) that calculates based on the fusion algorithm the depth map. After the positions and the sizes of the objects have became known, a smartphone (4) converts the information to a detectable feedback tone through a headphone (5), and, based on this tone, the person can recognize where the noise is coming from (i.e., position of the obstacle) and magnitude is the intensity (i.e., size of the obstacle). A power source (6) is also needed for the system to work.

### 5.2. Testing of the System

After the hardware implementation, to examine the working capabilities of the system, several measurements need to be done and also evaluated.

The evaluation should study the influence of all possibly important environmental and physical conditions, i.e.,

- influence of the number of the sensors
- influence of the measurement distance
- detection of small objects
- detection of several objects simultaneously
- detection of various materials
- detection of various shapes.

**Figure 11:**
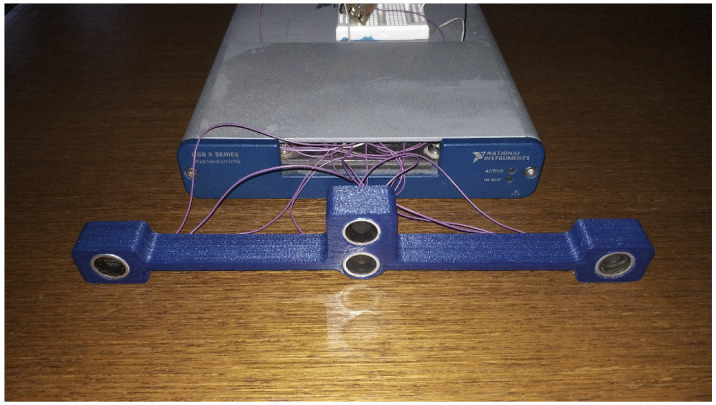
Test system built up with a National Instruments measuring unit.

For the measurement not a smartphone was used for the calculations, but an NI interface and Labview software for the faster debugging, and a PC to acquire the data live. To make the system fully portable and usable, to use an individually developed PCB is planned with a programable microcontroller i.e., PIC32MX695F512H from Microchip. In addition, more compact ultrasonic sensors are to be used like MEMS sensors i.e., SPU0410LR5H-QB. The measurement module is built up from one transmitter and three receivers as Figure 11 shows, and every measurement was accomplished with both three and two receiver setup. In case of every measurement, the evaluation area is 5 m × 5 m with a 1 cm resolution. The sensors can detect signals from as far as 8 m, but for higher distances than 6 m, the received signals are not always—for smaller obstacles—distinguishable enough from the electronic noise, and are thus not accurate enough to evaluate. The maximum distance can be extended with better amplifier circuits for the receivers or the signal output power of the transmitter has to be raised.

Based on the system needs, two assumptions could be considered. For the definition of the measured distance *l*, the refresh rate f=10 Hz is the speed of the subjective v=5 km/h =1.389 m/s, and the reaction time ( $t_{r}=1.1$ s) is needed. From these three conditions, the minimal distance is easily determinable shown as (10). Here, due to the safety reason, a safety factor $c_{f}=3$ was used

(10) $$\begin{array}{c} l=v\cdot \left(\frac{1}{f}+t_{r}\right)\cdot c_{f},\;l≃5m. \end{array}$$

The application of a safety factor was considered, because the experiments were static, whereas the device should be applicable in such an environment where both the user and the obstacles can move with small velocity. In the case of a dynamic environment, the changes in the frequency due to the Doppler effect should be considered as well. With a speed of 5 km/h of the user and 5 km/h of the approaching obstacle—another pedestrian—the maximal Doppler shift will be smaller than 1%, which has no practical effect on the operation.

**Figure 12:**
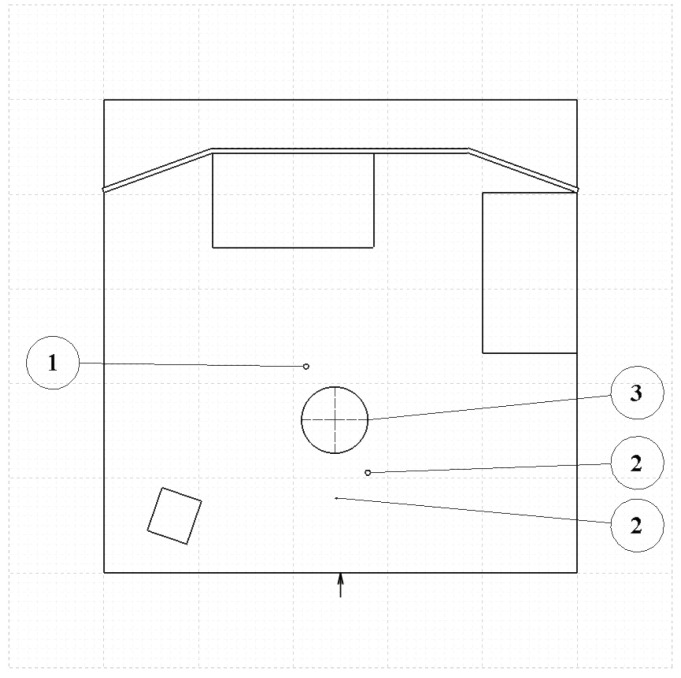
Test environment schematic plan, background grade shows the scale of the room: the distance between the thick lines is 1 m while the minor grid distance is 10 cm, the total scale of the room is 5 m × 5 m, the location of the device under testing is denoted by an arrow on the bottom of the plot, while the obstacle used in the measurement is numbered according to the test number.

In our case, the accuracy is determined by the sampling rate and the speed of the sound. The test system’s sampling rate for the whole system is $f_{s0}=500$ kHz, but the sampling rate is lower for the separate sensors. In the case of three sensors, the sampling rate is $f_{s}=166.67$ kHz, i.e., the third of the whole system’s $f_{s0}$ is due to the multiplexing. From this $f_{s}$, the sampling time $t_{s}=6\;\mu$s is given, which results in the resolution dl to be 2.04 mm if calculated with the nominal speed of the sound v=340 m/s. The second influencing parameter is the speed of the sound, which changes depending on the environment. In the realistic cases, this factor lies in the interval from v=325.4 m/s to v=351.2 m/s. This implies deviation from the nominal speed less than 5%. Projected to the border of the measured area with 5 m radius and taking into account the variation of the humidity and the temperature, the accuracy is ±25 cm. However, in realistic cases, the environment is constant or slowly changing, between two measurements; thus, practically, the accuracy is given solely by the sampling rate. Of course, the absolute measurements have still errors, but, in the relative cases, it does not affect the working of the system. A measurement environment is built up, based on the calculated limitations, which can be seen on Figure 12.

#### 5.2.1. Indoor Test No. 1

**Figure 13:**
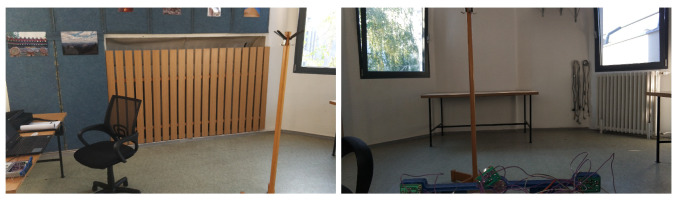
Indoor test No. 1.

During the first measurement, the test was carried out to verify whether the system works. The measured object was a coat hanger with the section size of 65 mm as it can be seen in Figure 13.

**Figure 14:**
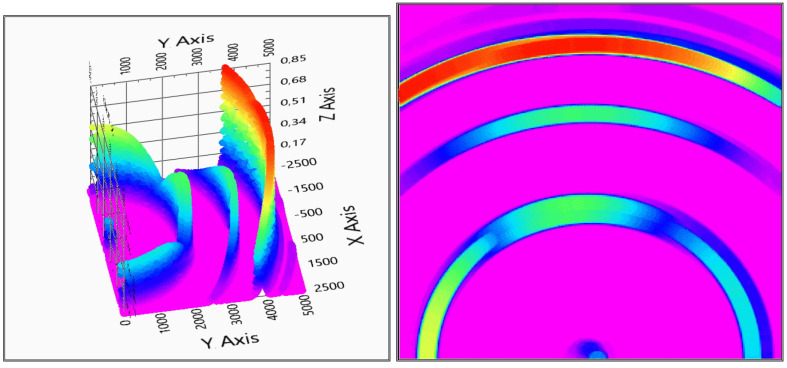
Evaluation of indoor test no.1 (2 receivers). In the right column, the top view is also shown (x,y: x,y space axis, *z*-axis: probability of solid object in x,y defined position).

**Figure 15:**
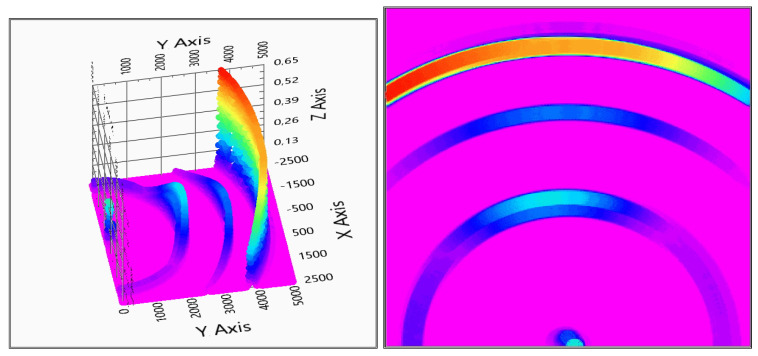
Evaluation of indoor test no.1 (3 receivers). In the right column, the top view is also shown (x,y: x,y space axis, *z*-axis: probability of solid object in an x,y defined position).

The measurement evaluation is easier if the graphic is viewed from above—top view—to get more adequate results of the positions. After the first system checking measurement, it can be concluded that the system can detect the measured object and gives back its position, see Figure 14 for the two receivers and Figure 15 for the three receivers measurements.

The evaluation shows that the system detects not only the coat hanger, but the table and the wall in the background too. It can be concluded that the measurement is influenced neither by the material nor the shape. The 5 m detection distance is feasible, as with a normal walking velocity of 5 km/h, the distance of 5 m is equal to 3.6 s time, thus the obstacle is detectable in time. In this case, the user has 3.6 s to detect the obstacle and avoid the collision.

There is a direct signal between the transmitter and the receivers, and, based on that fact that the evaluation diagrams show a ghost detection at coordinate (0,0), as it can be seen in Figure 14 and Figure 15.

With two receivers, the detection visualized in Figure 14 shows some ghosts on the depth map, but the detection is still usable. The three receivers’ solution of Figure 15 gives a more accurate measurement, but also the calculation time is increased with the third receiver data processing.

#### 5.2.2. Indoor Test No. 2

**Figure 16:**
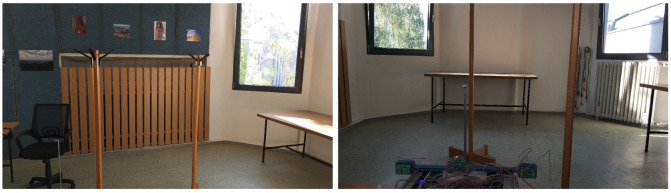
Indoor test No. 2.

In the second measurement, the sensitivity of detection and the ability of detecting multiple objects were tested. To simulate this case, a small round 5 mm diameter vertical metal rod was added, and another coat hanger to other position, while the original coat hanger remained in place—see Figure 16.

**Figure 17:**
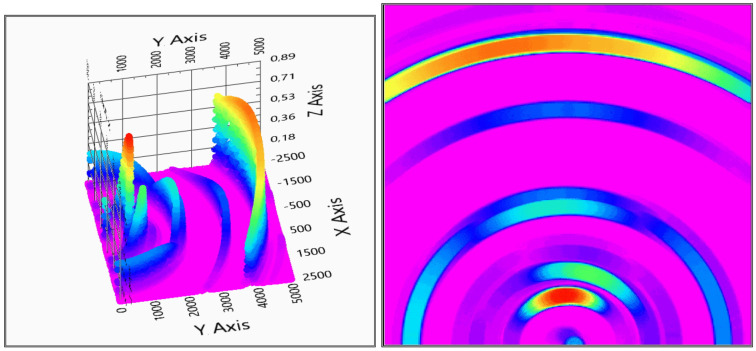
Evaluation of indoor test no.2 (2 receivers). In the right column, the top view is also shown (x,y: x,y space axis, *z*-axis: probability of solid object in x,y defined position).

**Figure 18:**
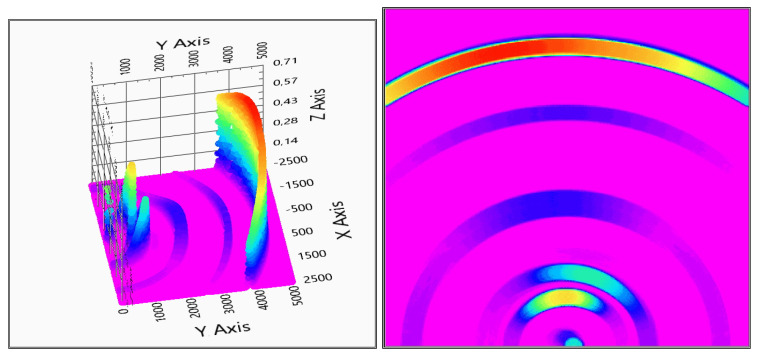
Evaluation of indoor test No. 2 (3 receivers). In the right column, the top view is also shown (x,y: x,y space axis, *z*-axis: probability of solid object in x,y defined position).

The comparison of two and three receivers are made with this layout too, and plotted in Figure 17 and Figure 18. From the figures, it is visible that, if there are more detectable objects, the system creates more ghost detections; like the shadows along the 1.5 m circle around the transmitter, the increases on the left and right-hand side of the coat hanger are at 2 m distance from the transmitter. In this case, the three receivers give a clearer picture. This was the point when it was decided that the third receiver is necessary to be added to the goggles, to have a more exact depth map from the environment. Because of the mathematical background, the three sensors measurement causes a lower possibility rate overall in the detection area, as it is visible in Figure 18.

Despite the lot of ghost detection, the multi object recognition is possible with the inverse mathematic fusion model (9).

#### 5.2.3. Indoor Test No. 3

**Figure 19:**
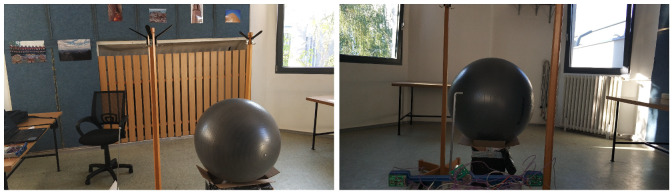
Indoor test No. 3.

The influence of various materials on the measurement was also examined. The layout was not changed; only a rubber ball was inserted between the two coat hangers, as shown in Figure 19.

**Figure 20:**
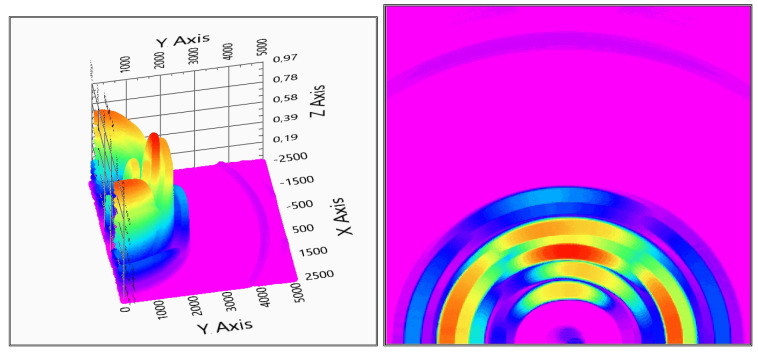
Evaluation of indoor test No. 3 (2 receivers). In the right column, the top view is also shown (x,y: x,y space axis, *z*-axis: probability of solid object in x,y defined position).

**Figure 21:**
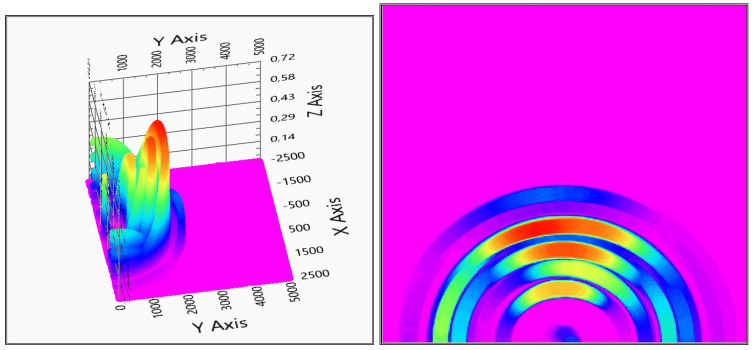
Evaluation of indoor test No. 3 (3 receivers). In the right column, the top view is also shown (x,y: x,y space axis, *z*-axis: probability of solid object in x,y defined position).

There were no real difference in the results between materials, and hard surfaced materials were inspected, like wood, metal, plastic, concrete. However, the rubber ball made a double echo—one from the front and one from the back side—see Figure 20 and Figure 21. The conclusions from this phenomenon are that the particular proportion of the ultrasonic wave can cross the rubber wall and reflect from the back wall of the ball, as it can be seen in Figure 20 and Figure 21. The measurement was also possible in the “shadow” of the ball, and, because the signals already crossed the ball, the sensing of the echo from the second coat hanger is significantly lower (Figure 20).

The most highlighted in this measurement is the difference between the number of used receivers. Because of the ghost detections with two receivers, the measurement is nearly not evaluable, while, in the three receiver case, the localization is acceptable for the proposals for aiding visually impaired people, in order to avoid obstacles.

## 6. Conclusions

A plan for a sensor device was proposed for helping visually impaired people in orientation. The device uses a single ultrasound source and two or three receivers attachable to a head set, like e.g., a pair of goggles.

The theoretical background of finding the obstacles is based on the time of flight method and uses sensor fusion. The algorithm determines the probability of the obstacle being present in each point of the scanned 3D space using a 4D matrix. This fourth dimension is composed of the three spatial coordinates and the sensor number as the fourth dimension, and, by the fusion, this fourth dimension is reduced.

The applicability of the method is demonstrated by measurements using a LabVIEW environment for both the two- and the three-sensor cases with a prototype. It is shown that the third sensor increases the precision and improves the results, and the third sensor makes the device applicable for real-life purposes.
